# Editorial: Translation of genetically engineered T cells in cancer immunotherapy

**DOI:** 10.3389/fimmu.2023.1278677

**Published:** 2023-09-07

**Authors:** Ralf-Holger Voss, Hakim Echchannaoui, He Huang, Shao-An Xue

**Affiliations:** ^1^ Institute of Immunology, Department of Research Center for Immunotherapy, Laboratory U. Sahin, University Medical Center (UMC) of the Johannes Gutenberg University, Mainz, Germany; ^2^ Department of Hematology and Medical Oncology, University Medical Center, Johannes Gutenberg-University, Mainz, Germany; ^3^ German Cancer Consortium partner site Frankfurt/Mainz, Mainz, Germany; ^4^ University Cancer Center Mainz, Mainz, Germany; ^5^ Institute of Hematology, Zhejiang University, Hangzhou, China; ^6^ Surgery, Medical School, Faculty of Medical Sciences, University College London, London, United Kingdom; ^7^ Genetic Engineering Laboratory, School of Biological & Environmental Engineering, Xi’An University, Xi’An, China

**Keywords:** genetic/cellular engineering, CAR-T, TCR-T, cancer immunotherapy, tumor specific/associated antigens, genome editing, immunosuppression, CAR toxicity

Genetically engineered T cells have made tremendous contributions to cancer immunotherapy. Chimeric antigen receptor (CAR)-modified T cells have demonstrated remarkable efficacy in hematological malignancies (1–5), however, clinical responses have not been convincing in solid tumors. T cell receptor (TCR)-engineered T cells showed promising results in some solid cancers (6–8), yet many hurdles remain to translate current genetically engineered T cells into more effective therapeutics, to achieve higher and durable clinical responses.

In this Research Topic, we compile recent advances in T cell immunotherapy, including the identification of new promising antigens, the optimisation of genetically modified TCR- and CAR- T cells to improve their persistence and reduce their toxicity. We also discuss strategies to overcome the suppressive tumor microenvironment (TME) and perspectives in T cell manufacturing.

## 1 Identification and validation of novel antigens for cancer immunotherapy

In recent years, tumor-specific mutated antigens, also called neoantigens (neoAgs) have emerged as a promising class of immunogenic antigens for immunotherapy (9). These antigens are exclusively expressed and presented on tumor cells, and represent an attractive therapeutic tool for solid tumors, in particular for TCR-engineered T cells (10).

In this Research Topic, Immisch et al. identified a neoepitope comprising Rac1P29S amino acid mutation, which is the third most common hotspot mutation in melanoma. They have not only isolated and characterised TCRs that can recognise this HLA-A*02:01-binding neoepitope, but also demonstrated TCR-T induced cytotoxicity against Rac1P29S expressing cancer cells *in vitro* and *in vivo* after adoptive T cell therapy.

Although neoAgs are considered as truly tumor-specific antigens (11), Amerongen et al. pursue the concept of identifying highly expressed tumor-associated antigens in ovarian cancer from pooled mRNAseq data bases, and a reverse immunology approach for the most prevalent HLA restriction elements. The candidate antigens comprising PRAME, CTCFL and CLDN6 exhibited a 20-fold higher expression in tumors compared to normal cells. High-avidity TCRs were isolated, cloned and characterised *in vitro* to exclude potential on/off-target-reactivities, and demonstrated potent antitumor activities, making these TCRs especially useful for adoptive therapy in ovarian cancers, generally considered as ‘cold tumors’ with less T-cell infiltrates.

## 2 Strategies to enhance expression, specificity, affinity, and (signaling) functions of the engineered molecules

Barden et al. addressed the issue of cross-activation of a CAR with the endogenous TCR in CAR-T cells. They found out that the antigen-dependent activation of T-cells by the triggered immunoreceptor (IR) exclusively results in phosphorylation of the CAR/CD3ζ or TCR/CD3ζ, respectively, thus excluding reciprocal cross-activation. This is in line with elaborate microscopy analyses elucidating their mutual spatial exclusion upon either IR activation. However, TCRs and CARs can co-operate by means of antigen recognition by the endogenous TCR and costimulation by CD28 incorporated in CD28/ζ CARs. Collectively, the authors claim that TCR/CD28 CAR-signaling may be exploited for Boolean logic “AND” gating (12), stressing the importance of endogenous TCRs for providing a non-TME tonic signaling for CAR-T persistence in patients.

TCR-based bispecific T cell engagers (TCE) are emerging therapeutics as recently reported by positive phase III clinical results of a gp100/HLA-A2-TCR/anti-CD3 bifunctional in melanoma patients (13). Unlike antibody-based TCE (14), which have been widely studied, little is known about how the formats of TCR-based TCE affect their potencies. In this context, Van Diest et al. recently developed a novel TCE format based on the soluble γ9δ2TCR-antiCD3 bispecific molecule (GAB) (15), and described an alternative design based on the multimerisation of GABs to improve their potency. Here, van Diest et al. could further enhance the fraction of GAB-dimers by shortening the linker length within the anti-CD3 scFv, and showed that GAB-dimers were superior in function, without apparent on-target/off-tumor reactivity.

## 3 Novel strategies for genetic engineering of T cells, including CAR-T and TCR-T cells

Hiltensperger and Krackhardt have reviewed in depth the TCR-T and CAR-T field, comprising several aspects, from the design of different generations of CARs for providing signal 1 to 3 in T-cell activation, prevention of TCR mispairing by TCR protein engineering and genome editing, the set up of allogeneic T-cell transfer to boost T-cell fitness and T-cell graft manufacturing, adverse events to reckon with and counteracting tumor escape, persistence of T-cells, to treatment modalities for solid cancers. The review also covers gene transfer shuttle systems, persistence of T-cells, gene delivery approaches *in vivo*, potentially arising adverse events such as on/off-target toxicities, and cellular and molecular strategies to force back tumor escape mechanisms and resistance of solid tumors in TME, and provides an overview of ongoing clinical trials.

Degirmencay et al. have explored how modifications of framework residues in the TCR variable domains affect TCR expression and function. They used bioinformatic and protein structural analyses to identify candidate amino acid residues in the framework of the variable β domain predicted to drive high TCR surface expression. Replacing these residues in poorly expressed TCRs resulted in improved surface expression and enhanced target specific killing by these engineered TCR-T. Their results corroborated improved expression and functionality, while at the same time reducing the risk of toxicity associated with TCR mispairing.

Autologous T cell engineering is not only costly but also time-consuming, limiting the number of patients who can benefit from this new therapy. Yu et al. developed an allogeneic approach by generating CD19-CAR T cells from cord blood of allogeneic donor, and demonstrated their anti-tumor activity *in vitro* and *in vivo* using a diffuse large B-cell lymphoma model (DLBCL). The rationale behind this idea is the higher proportion of naïve T-cells from cord blood that can be redirected into potent effector T cells, thus having a better anti-tumor activity. This strategy may provide a broader option for immunotherapy, offering readily accessible “off-the-shelf” cellular products.

## 4 Strategies for overcoming the immunosuppressive tumor microenvironment

In a previous study, TCR-T cells were engineered to disrupt PD-1 upregulation upon antigen encounter by CRISPR/Cas9 genome editing to minimise immunosuppression through PD-L1-positive tumor cells (16). Here, Kim et al. went on one step further and took advantage of the tightly regulated PDCD1 locus for replacing PD-1 by a pleiotropic cytokine such as IL-12 to positively affect the persistence of T-cells in TME. By this means, an inhibitory signal of PD-1 would be reversibly inverted into a stimulatory signal of IL-12 only in the presence of the tumor antigen recognised by the introduced TCR, and hence in a local (TME) and timely (as long as antigen is present) restricted manner so as to avert cytokine-induced toxicities.

## 5 Manufacturing platforms for vector production and cellular engineering

Niu et al. analysed the phenotype of lentivirally (LV) transduced versus PiggyBac transposon (PB)-transfected CAR-T cells *in vitro* and *in vivo*. They scrutinised biomarker expression rates and phenotype (effector versus central memory subsets), cytokine/chemokine secretions and cytolyses, including a transcriptomic approach, and validation of an *in vivo* tumor model. In PB- compared to LV-CAR-T, they reported on higher expression of IL-9, a cytokine that enhances anti-tumor responses, and on lower expression of IL-6, the hub cytokine triggering cytokine release syndrome, suggesting a favourable profile of the former manufacturing platform. Importantly, both systems control tumor cells comparably *in vivo*, in line with recently published work (17).

**Figure 1:**
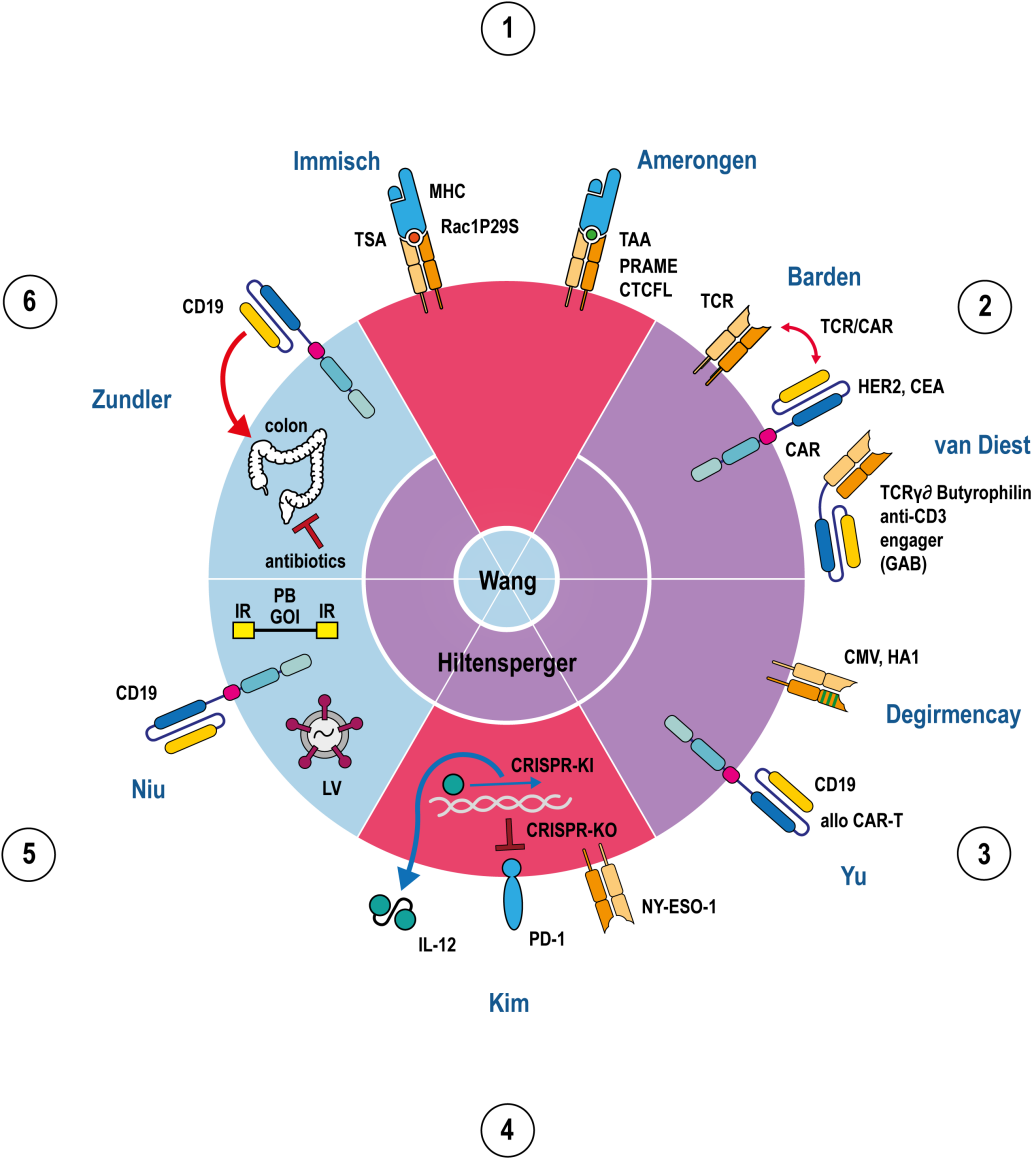
Apple pie chart attributing 11 articles (2 reviews, 8 original research articles, 1 case report) to the 6 topics 1.-6. listed in the Editorial summary in a clockwise manner. The 11 articles are indicated by their first author names. The 6 apple pie pieces are colour-coded according to their assigned articles covering either the subject TCR (red), CAR (blue), or both (purple). The review articles are shown in the center of the chart indicating to which topics they refer to. TCRs, CARs, MHCs, PD-1, IL-12, and GABs are depicted in a stylised fashion. The outline of the circle represents the T-cell membrane where all immunoreceptors (TCRs, CARs) are embedded. IL-12, GABs and MHCs are either soluble molecules or membrane proteins from target cells, respectively, and hence located outside the circle. Tumor antigens presented by MHC are indicated as coloured spheres, depending on the particular peptide. Antigen specificities of TCRs, CARs and GABs are denoted besides the stylised molecule. Helical DNA, lentivirus, recombinant transposon DNA, and colon are adumbrated as cell or body internal components and hence, located inside the circle. Red double/single arrows represent mutually interacting/inflammatory, a ‘_┴_’ inhibitory reactions, a blue arrow an activating reaction in transcription/translation, green lines in a TCR denotes point mutations in TCR Vβ domain. Abbreviations: TSA tumor specific antigen, TAA tumor associated antigen, TCR T-cell receptor, CAR chimeric antigen receptor, GAB gamma delta TCR anti-CD3 bispecific molecule, CMV cytomegalovirus, allo alloreactive, IL-12 interleukin 12, KO knock out, KI knock in, CRISPR clustered regularly interspaced short palindromic repeats, PD-1 programmed cell death protein 1, LV lentivirus, PB PiggyBac, IR inverted repeats, GOI gene of interest, Rac1P29S, PRAME, CTCFL, Her2, CEA, CMV, HA1, NY-ESO-1, CD19 represent processed or full length tumor/viral antigens, respectively.

## 6 Strategies for reducing toxicity and improving safety

Although, CAR-T cell therapy approximates a consolidated mainstay in the treatment of several hematologic malignancies, adverse side effects may occur. In this Research Topic, Wang et al. reviewed CAR-T treatments in the context of multiple myeloma and potential toxicities, while Hiltensperger & Krackhardt covered CAR-T and TCR-T approaches in general, highlighting recent innovations capable of enhancing efficacy and reducing toxicity. Zundler et al. also reported a case of a rare complication in a patient treated with CD19 CAR-T for DLBCL, who developed chronic diarrhea with characteristics of inflammatory bowel disease-like colitis. Resolution of colitis occurred by using an antibiotic therapy that might have changed the intestinal microbiota, which most likely limited the stimulation of these intestinal infiltrating CAR-T cells.

## Author contributions

RHV: Writing – review & editing, Conceptualization, Writing – original draft. HE: Writing – review & editing. HH: Writing – review & editing. SAX: Writing – original draft, Writing – review & editing, Conceptualization.

## References

[1] Kalos M Levine BL Porter DL Katz S Grupp SA Bagg A. T cells with chimeric antigen receptors have potent antitumor effects and can establish memory in patients with advanced leukemia. Sci Transl Med (2011) 3(95):95ra73. doi: 10.1126/scitranslmed.3002842 PMC3393096 21832238

[2] Grupp SA Kalos M Barrett D Aplenc R Porter DL Rheingold SR. Chimeric antigen receptor-modified T cells for acute lymphoid leukemia. New Engl J Med (2013) 368(16):1509–18. doi: 10.1056/NEJMoa1215134 PMC4058440 23527958

[3] Maude SL Laetsch TW Buechner J Rives S Boyer M Bittencourt H. Tisagenlecleucel in children and young adults with B-cell lymphoblastic leukemia. New Engl J Med (2018) 378(5):439–48. doi: 10.1056/NEJMoa1709866 PMC5996391 29385370

[4] Boyiadzis MM Dhodapkar MV Brentjens RJ Kochenderfer JN Neelapu SS Maus MV. Chimeric antigen receptor (CAR) T therapies for the treatment of hematologic Malignancies: clinical perspective and significance. J Immunother Cancer (2018) 6(1):137. doi: 10.1186/s40425-018-0460-5 30514386 PMC6278156

[5] Ruella M Maus MV. Catch me if you can: Leukemia Escape after CD19-Directed T Cell Immunotherapies. Comput Struct Biotechnol J (2016) 14:357–62. doi: 10.1016/j.csbj.2016.09.003 PMC5061074 27761200

[6] Robbins PF Kassim SH Tran TL Crystal JS Morgan RA Feldman SA. A pilot trial using lymphocytes genetically engineered with an NY-ESO-1-reactive T-cell receptor: long-term follow-up and correlates with response. Clin Cancer Res (2015) 21(5):1019–27. doi: 10.1158/1078-0432.CCR-14-2708 PMC4361810 25538264

[7] D’Angelo SP Melchiori L Merchant MS Bernstein D Glod J Kaplan R. Antitumor activity associated with prolonged persistence of adoptively transferred NY-ESO-1 (c259)T cells in synovial sarcoma. Cancer Discov (2018) 8(8):944–57. doi: 10.1158/2159-8290.CD-17-1417 PMC8092079 29891538

[8] Nagarsheth NB Norberg SM Sinkoe AL Adhikary S Meyer TJ Lack JB. TCR-engineered T cells targeting E7 for patients with metastatic HPV-associated epithelial cancers. Nat Med (2021) 27(3):419–25. doi: 10.1038/s41591-020-01225-1 PMC9620481 33558725

[9] Schumacher TN Schreiber RD. Neoantigens in cancer immunotherapy. Science (2015) 348(6230):69–74. doi: 10.1126/science.aaa4971 25838375

[10] Garber K. Driving T-cell immunotherapy to solid tumors. Nat Biotechnol (2018) 36(3):215–9. doi: 10.1038/nbt.4090 29509745

[11] Coulie PG Van den Eynde BJ van der Bruggen P Boon T. Tumour antigens recognized by T lymphocytes: at the core of cancer immunotherapy. Nat Rev Cancer (2014) 14(2):135–46. doi: 10.1038/nrc3670 24457417

[12] Hamieh M Mansilla-Soto J Riviere I Sadelain M. Programming CAR T cell tumor recognition: tuned antigen sensing and logic gating. Cancer Discov (2023) 13(4):829–43. doi: 10.1158/2159-8290.CD-23-0101 PMC10068450 36961206

[13] Nathan P Hassel JC Rutkowski P Baurain JF Butler MO Schlaak M. Overall survival benefit with tebentafusp in metastatic uveal melanoma. N Engl J Med (2021) 385(13):1196–206. doi: 10.1056/NEJMoa2103485 34551229

[14] van de Donk N Zweegman S. T-cell-engaging bispecific antibodies in cancer. Lancet (2023) 402(10396):142–58. doi: 10.1016/S0140-6736(23)00521-4 37271153

[15] van Diest E Hernandez Lopez P Meringa AD Vyborova A Karaiskaki F Heijhuurs S. Gamma delta TCR anti-CD3 bispecific molecules (GABs) as novel immunotherapeutic compounds. J Immunother Cancer (2021) 9(11):e003850. doi: 10.1136/jitc-2021-003850 34815357 PMC8611453

[16] Stadtmauer EA Fraietta JA Davis MM Cohen AD Weber KL Lancaster E. CRISPR-engineered T cells in patients with refractory cancer. Science (2020) 367(6481):eaba7365. doi: 10.1126/science.aba7365 32029687 PMC11249135

[17] Lin Z Liu X Liu T Gao H Wang S Zhu X. Evaluation of Nonviral piggyBac and lentiviral Vector in Functions of CD19chimeric Antigen Receptor T Cells and Their Antitumor Activity for CD19(+) Tumor Cells. Front Immunol (2021) 12:802705. doi: 10.3389/fimmu.2021.802705 35082789 PMC8784881

